# Effect of tadalafil combined with atorvastatin on hemodynamics and sexual function in middle-aged and elderly patients with hyperlipidemia complicated with erectile dysfunction

**DOI:** 10.12669/pjms.37.7.4257

**Published:** 2021

**Authors:** Lei Du, Jiang-hua Jia, Wen-yong Xue, Jin-chun Qi

**Affiliations:** 1 Lei Du, Department of Urology, The Second Hospital of Hebei Medical University, No. 215 Heping Xi Road, Shijiazhuang, Hebei, China; 2 Jiang-hua Jia, Department of Urology, The Second Hospital of Hebei Medical University, No. 215 Heping Xi Road, Shijiazhuang, Hebei, China; 3 Wen-yong Xue, Department of Urology, The Second Hospital of Hebei Medical University, No. 215 Heping Xi Road, Shijiazhuang, Hebei, China; 4 Jin-chun Qi, Department of Urology, The Second Hospital of Hebei Medical University, No. 215 Heping Xi Road, Shijiazhuang, Hebei, China

**Keywords:** Atorvastatin, Erectile dysfunction, Hemodynamics, Hyperlipidemia, Tadalafil, Treatment

## Abstract

**Objective:**

To evaluate the effect and clinical significance of tadalafil combined with atorvastatin on hemodynamics and sexual function in middle-aged and elderly patients with hyperlipidemia complicated with Erectile dysfunction (ED).

**Methods:**

Eighty patients with hyperlipidemia complicated with ED who were treated at The Second Hospital of Hebei Medical University from January 2019 to June 2020 were selected. Using a completely randomized design experimental method, these 80 patients were randomly divided into two groups: the experimental group and the control group, with 40 cases in each group. The control group was treated with a single drug, atorvastatin calcium, while the experimental group was given tadalafil orally on the basis of the control group for 3 months. Changes in the levels of inflammatory factors such as IL-6, TNF and CRP, adverse drug reactions, changes in hemodynamic indicators such as HSV, LSV, PSV, HCT and ESR before and after treatment, as well as changes in sexual function after treatment were compared and analyzed between the two groups.

**Results:**

TNF-a, CRP and IL-6 in the experimental group were significantly lower than those in the control group after treatment, with statistically significant differences (p<0.05). There was no significant difference in the incidence of adverse drug reactions between the two groups (p=0.18). After treatment, hemodynamic indexes and sexual function indexes of the experimental group were significantly improved compared with those in the control group, with statistically significant differences (p<0.05).

**Conclusion:**

A significant improvement effect can be achieved by tadalafil combined with atorvastatin on hemodynamics and sexual function in middle-aged and elderly patients with hyperlipidemia complicated with ED. At the same time, the combination of the two has synergism on inflammatory factors and blood rheology, and the incidence of adverse reactions is not significantly increased.

**Abbreviations:**
ED: Erectile dysfunction,
PDE-5: Phosphodiesterase-5,
IL-6: Interleukin- 6,
CRP: C-reactionprotein,
HSV: High shear viscosity,
LSV: Low cut viscosity of whole blood,
PSV: The plasma viscosity,
HCT: Hematocrit,
ESR: Erythrocyte sedimentation rate,
IIEF-5: International Index of Erectile Function Questionnaire – 5,
HMG-CoA: Hydroxy-methylglutaryl coenzyme A,
cGMP: cyclic Guanosinc monophosphate,
TNF-α: Tumor necrosis factor-α,
IFN-γ: Interferon-γ,
TC: Total cholesterol,
TG: Triglycerides,
HDL-C: High density lipoprotein cholesterol.

## INTRODUCTION

Erectile dysfunction (ED) is prevalent in middle-aged and elderly people, and its incidence has been increasing year by year in recent years.^1^ Data show that currently about 150 million middle-aged and elderly people worldwide suffer from ED, causing serious impacts on the quality of life of middle-aged and elderly men.^2^ Studies have shown that^3^ ED is a multi-factor disease characterized by multiple etiology and complex pathogenesis. Among various etiologies, hyperlipidemia is one of the main causes of ED in middle-aged and elderly men. It not only endangers the physical and mental health of patients, but also causes other system or organ diseases, such as cardiovascular and cerebrovascular complications, which further adversely affects the health of patients.^4^ Certain benefits can be exerted on ED by improving blood lipid levels. PDE-5 (phosphodiesterase) inhibitors are characterized by various effects such as protecting or enhancing vascular endothelial function and improving hemodynamics.^5^ It was considered in the study of Lee et al.^6^ that hyperlipidemia was associated with the risk of ED, and tadalafil, a new generation of PDE-5 (phosphodiesterase) inhibitor, had a certain synergistic effect on the improvement of hemodynamics in patients with hyperlipidemia.

In this study, atorvastatin calcium combined with tadalafil was used in the treatment of middle-aged and elderly patients with hyperlipidemia complicated with ED. The hemodynamics and erectile function of the patients were significantly improved, with no significant increase in side effects, and certain curative effect was achieved. The specific details are now reported as follows.

## METHODS

### Ethical approval

The study was approved by the Institutional Ethics Committee of The Second Hospital of Hebei Medical University at June 4, 2020, and written informed consent was obtained from all participants.

### Patient information:

### Case Inclusion Criteria:

1. Patients who meet the diagnostic criteria of hyperlipidemia with TC≥5.65mmol/L, TG≥1.7mmol/L and HDL-C<0.91mmol/L.^7^
2. Patients with mild to moderate ED (with IIEF-5 score of 8-21).^8^
3. Patients aged 50-65 years old.
4. Patients whose family members are willing and able to cooperate to complete the study and have good treatment compliance.
5. Patients without contraindications to the drugs used in this study.
6. Patients who signed the informed consent.

### Case Exclusion Criteria:

1. Patients with allergy to the drugs involved in this study.
2. Patients with cardiovascular, cerebrovascular diseases and diabetes.
3. Patients with mental or cognitive dysfunction who cannot cooperate with the completion of the study.
4. Patients who have taken other lipid-lowering drugs or other PDE-5 inhibitors in the last 2 weeks.
5. Patients who have taken relevant drugs affecting the study such as immunosuppressants and hormones in the near future.

Eighty patients with hyperlipidemia complicated with ED who were treated at The Second Hospital of Hebei Medical University from January 2019 to June 2020 were selected. Using a completely randomized design experimental method, these 80 patients were randomly divided into two groups, with 40 cases in each group. Patients in the experimental group ranged in age from 53 - 65 years old, with an average of 60.32±3.50 years old, while those in the control group ranged from 51 - 63 years old, with an average of 59.25±3.36 years old. There was no significant difference in general data between the two groups, which were comparable (Table-I).

**Table I:** Comparative analysis of general data between the experimental group and the control group (X¯±S) n=40.

*Indicators*	*Experimental group*	*Control group*	*t/χ^2^*	*p*
Age	60.32±3.50	59.25±3.36	1.39	0.67
IIEF-5 score	15.16±3.02	14.84±2.44	0.52	0.60
Blood lipid level				
TC(mmol / L)	5.94±0.83	5.91±0.79	0.17	0.86
TG(mmol / L)	2.83±0.47	2.79±0.36	0.43	0.67
HDL-C(mmol / L)	0.71±0.06	0.69±0.08	1.26	0.21

P>0.05.

### Treatment methods:

Patients in both groups received general treatment, including a light diet, moderate exercise, improvement of unhealthy lifestyle habits such as drinking, smoking, staying up late and so on. The control group was given atorvastatin calcium 20 mg once a day orally at bedtime or 3 hours after dinner. In contrast, the experimental group was given tadalafil 10 mg orally once a day^9^ on the basis of the control group for 3 months.

### Observation indicators:

### 1. Comparative analysis of changes in inflammatory factors:

the changes in levels of inflammatory factors such as IL-6, TNF and CRP were observed in the study group and control group before and after treatment.

### 2. Comparative analysis of adverse drug reactions of the two groups:

adverse drug reactions in the two groups within 1 month after medication were recorded, including muscle pain, nasopharyngeal congestion, facial flushing, digestive tract reaction, etc.

### 3. Hemodynamic changes:

the changes of hemodynamic indicators, such as HSV, LSV, PSV, HCT and ESR, in the experimental group and the control group before and after treatment were compared and analyzed.

### 4. Assessment of sexual function changes:

including the assessment of the sexual function changes in the two groups after treatment: the International Index of Erectile Function Questionnaire - 5 (IIEF-5) was used to conduct a questionnaire survey on the patients, and the scores obtained were added together, a score ≥ 22 was considered as normal erectile function, 12 to 21 were classified as mild ED, 8 to 11 were classified as moderate ED, 5 to 7 are classified as severe ED. Nocturnal penile tumescence test: patients underwent at least 2 - 3 consecutive nights of the test, slept at least 5h per night, and avoided alcohol and sleeping pills for 2 nights prior to the test. The main measurement parameters include the number of penile erections at night, the total duration of erection maintenance, and the duration of penile root hardness greater than 60%.^10^

### Statistical analysis

All the data were statistically analyzed by SPSS 20.0 software, and the measurement data were expressed as (±s). Two independent sample t-test was used for inter-group data analysis, paired t test was used for intra-group data analysis, and χ^2^t was adopted for rate comparison. p<0.05 indicates a statistically significant difference.

## RESULTS

### Comparative analysis of changes in inflammatory factors before and after treatment between the two groups

The changes in inflammatory factors before and after treatment between the two groups are shown in Table-II, indicating that TNF-a, CRP, IL-6 and other inflammatory factors in the two groups were significantly increased before treatment, with no significant difference (p>0.05). The above indicators were decreased after treatment, with a statistically significant difference (p<0.05). TNF-a, CRP and IL-6 in the experimental group were significantly lower than those in the control group after treatment, with statistically significant differences (TNF-a, p=0.01, CRP, p=0.00, IL-6, p=0.03).

**Table II:** Comparative analysis of changes in inflammatory factors before and after treatment between the two groups (X¯±S) n=40.

*Group*		Before treatment*	*After treatment*	*t*	*p*
TNF-ɑ(ng/L)	Experimental groupΔ	44.73±9.75	23.310±8.15	20.21	0.00
	Control groupΔ	44.68±10.01	26.45±8.57	17.26	0.00
	t	0.36	2.49	
	p	0.21	0.01	
CRP(mg/L)	Experimental groupΔ	80.46±15.61	15.13±3.35	25.12	0.00
	Control groupΔ	81.02±16.27	20.03±3.41	23.21	0.00
	t	0.07	5.53		
	p	0.83	0.00		
IL-6(ng/L)	Experimental groupΔ	16.23±5.46	9.37±1.24	16.03	0.00
	Control groupΔ	16.25±5.81	12.58±3.65	15.05	0.00
	t	0.58	5.21		
	p	0.26	0.03		

* p>0.05, Δp<0.05

### Comparative analysis of adverse drug reactions between the two groups

The incidence of adverse drug reactions in the two groups after treatment was compared and analyzed, suggesting that the incidence of adverse reactions in the experimental group was 30%, and that in the control group was 17.5%. The incidence of adverse reactions in the experimental group was higher than that in the control group, but there was no statistical significance (p=0.18). (Table-III).

**Table III:** Comparative analysis of adverse drug reactions of the two groups after treatment (X¯±S) n=40.

*Group*	*Muscle pain*	*Gastrointestinal reaction*	*Mucosal hyperemia*	*Allergy*	*Incidence*
Experimental group	5	2	3	2	12(30%)
Control group	3	0	0	4	7(17.5%)
χ^2^					1.72
p					0.18

p<0.05.

### Comparative analysis of hemodynamic levels between the two groups before and after treatment

The hemodynamic changes of the two groups before and after treatment are shown in (Table-IV), suggesting that the indicators such as HSV, LSV, PSV, HCT and ESR in the two groups were significantly increased before treatment, with no significant difference (p>0.05). The above indicators were decreased after treatment, with statistically significant differences (p<0.05). The levels of HSV, LSV, PSV, HCT and ESR in the experimental group were significantly lower than those in the control group after treatment, with statistically significant differences (HSV, LSV, PSV, HCT and ESR, p=0.00) (Table-IV).

**Table IV:** Comparison and analysis of hemodynamic levels between the two groups before and after treatment (X¯±S) n=40.

*Group*		Before treatment*	*After treatment*	*t*	*p*
HSV(mPa*s)	Experimental groupΔ	7.85±1.07	4.73±0.82	14.63	0.00
	Control groupΔ	7.92±1.15	6.03±0.76	8.67	0.00
	t	0.28	7.35		
	p	0.77	0.00		
LSV(mPa*s)	Experimental groupΔ	18.25±6.73	11.13±3.46	5.95	0.00
	Control groupΔ	17.96±7.03	15.08±3.58	2.31	0.02
	t	0.18	5.02		
	p	0.85	0.00		
PSV(mPa*s)	Experimental groupΔ	3.89±1.07	2.37±0.24	8.77	0.00
	Control groupΔ	3.85±1.02	1.18±0.65	13.91	0.00
	t	0.17	10.86		
	p	0.86	0.00		
HCT(%)	Experimental groupΔ	47.06±8.34	33.58±4.77	8.87	0.00
	Control groupΔ	46.85±7.97	42.01±6.31	3.01	0.00
	t	0.12	6.74		
	p	0.91	0.00		
ESR(mm*h^-1^)	Experimental groupΔ	16.08±5.73	4.87±2.06	11.64	0.00
	Control groupΔ	15.74±6.01	9.07±2.33	6.54	0.00
	t	0.26	8.54		
	p	0.80	0.00		

* p>0.05, Δp<0.05

### Comparative analysis of the improvement of sexual function between the two groups

After treatment, the sexual function of the experimental group improved significantly, which was characterized by superior IIEF-5 score, nocturnal penile erection times, total erection duration, penile root hardness > 60% maintenance time compared with the control group, with statistically significant differences (p=0.00) (Table-V).

**Table V:** Comparative analysis of the improvement of sexual function between the two groups (X¯±S) n=40.

*Indicators*	*Experimental group*	*Control group*	*t*	*p*
IIEF-5	19.27±2.35	15.07±1.92	8.75	0.00
Times of erections (times)	4.57±1.66	3.83±1.12	5.49	0.00
Total erection time (min)	65.73±10.61	57.58±11.24	7.43	0.00
Penile root hardness > 60% maintenance time (min)	43.38±6.92	37.41±9.67	8.49	0.00

p<0.05.

## DISCUSSION

ED is a common andrological disease with complex etiology. Metabolic diseases such as hyperlipidemia, diabetes, and hypertension are the most common risk factors for ED in middle aged and elderly people.^11^ ED is also considered as a silent warning signal of vascular disease.^12^ Studies have shown^13^ that the proportion of patients with hyperlipidemia in all patients with ED is about 41.8%. The increase in TC levels and the decrease in HDL are important risk factors for ED. For every 1 mmol/L increase in TC, the risk of ED increases by 1.32 times, while for every 1 mmol/L increase in HDL, the risk of ED decreases by 0.38 times, suggesting that the severity of hyperlipidemia has a close bearing on the occurrence of ED. It is still unclear under what mechanism hyperlipidemia leads to the occurrence of ED, but it is generally believed that insufficient blood supply to the penile artery caused by atherosclerosis and inflammation caused by hyperlipidemia are important mechanisms of ED occurrence.^14^

Atorvastatin is a hydroxy-methylglutaryl coenzyme A (HMG-CoA) inhibitor commonly used clinically as a lipid-lowering drug. Relevant studies^15^ suggest that atorvastatin lipid-lowering drugs can exert its unique role in improving erectile function for patients with ED caused by hyperlipidemia as the only risk factor. However, patients with ED caused by a single factor are rarely seen clinically due to the more complex etiology and pathogenesis of ED. Consequently, a limited therapeutic effect will be achieved by a single drug. On the contrary, the combination of drugs with different mechanisms of action may play a more ideal therapeutic effect for hyperlipidemia ED.^16^

Currently, phosphodiesterase-5 (PDE-5) inhibitors are used clinically as the first-line therapy for ED. Sildenafil is a drug commonly used clinically for ED, but it has a greater impact on the cardiovascular system, especially in elderly patients, with obvious side effects. In contrast, tadalafil, as a new-generation PDE-5 inhibitor, has similar efficacy and higher safety with sildenafil. More than that, long-term oral administration of tadalafil in small doses can significantly improve vascular endothelial function, increase vascular elasticity, and better improve hemodynamic indicators.^17^ In view of this, tadalafil is considered a better option for ED treatment.^18^ It is considered by Brock et al.^19^ that with tadalafil, erectile function in all clinical populations could be effectively improved. Tadalafil, taken orally once daily at a low dose, is safer and more effective than taking high doses when necessary, especially in patients with diabetes, hypertension, hyperlipidemia and alcohol consumption.

Chronic low-grade inflammation characterized by metabolic syndrome may further aggravate the unsmoothness of local arteriosclerotic vascular endothelium caused by hyperlipidemia^20^ and may lead to local vascular fibrosis,^21^ further aggravating the local ischemic state. The results show that,^22^ in addition to vasodilation and anti-fibrotic proliferation, PDE-5 inhibitors also exert a direct anti-inflammatory effect by increasing the content of cGMP. It binds to the cGMP site, reduces the content of TNF-α, and shows a tendency to decrease IFN-γ.^23^ According to the research carried out by Elbardisy,^24^ certain therapeutic effects can be achieved by intranasal application of tadalafil, such as significantly reducing the content of inflammatory factors in serum of subjects, reducing inflammation and oxidative stress, and alleviating various biochemical indicators of patients with metabolic diseases.^25^

It is shown in our study results that TNF-a, CRP and IL-6 in the experimental group are significantly lower than those in the control group after treatment, with statistically significant differences (TNF-a, p=0.01, CRP, p=0.00, IL-6, p=0.03). The incidence of adverse drug reactions in the two groups after treatment is compared and analyzed, suggesting that the incidence of adverse reactions in the experimental group is 30%, and that in the control group is 17.5%. The incidence of adverse reactions in the experimental group is higher than that in the control group, but there is no statistical significance (p=0.18). The hemodynamic indicators such as HSV, LSV, PSV, HCT and ESR in the experimental group after treatment are significantly improved compared with those in the control group, with a statistically significant difference (p=0.00). After treatment, the sexual function of the experimental group improved significantly, which is characterized by superior IIEF -5 score, nocturnal penile erection times, total erection duration, penile root hardness > 60% maintenance time compared with the control group, with statistically significant differences (p=0.00).

### Limiations of the study

It includes small sample size and short follow-up time. In addition, only patients with mild to moderate ED are selected as subjects to ensure the effectiveness of the study. Based on this, relevant countermeasures are being carried out to actively enrich the sample content, further extend the time of follow-up, and classify different types of erectile dysfunction more precisely and further include patients with severe ED in the study, so as to conduct a more objective evaluation of the efficacy of this treatment regimen for patients with different severity and disparate types of ED complicated with hyperlipidemia.

## CONCLUSION

Significant improvement can be achieved by tadalafil combined with atorvastatin on hemodynamics and sexual function in middle-aged and elderly patients with hyperlipidemia complicated with ED. At the same time, the combination of the two has synergism on inflammatory factors and blood rheology, and the incidence of adverse reactions is not significantly increased.

### Authors’ Contributions:

**LD** **and** **JQ** designed this study and prepared this manuscript, and are responsible and accountable for the accuracy or integrity of the work.

**JJ** collected and analyzed clinical data.

**WX** significantly revised this manuscript.
